# Identification and characterization of a highly motile and antibiotic refractory subpopulation involved in the expansion of swarming colonies of Paenibacillus vortex

**DOI:** 10.1111/1462-2920.12160

**Published:** 2013-06-14

**Authors:** Dalit Roth, Alin Finkelshtein, Colin Ingham, Yael Helman, Alexandra Sirota-Madi, Leonid Brodsky, Eshel Ben-Jacob

**Affiliations:** 1 The Sackler School of Medicine, Tel Aviv UniversityTel Aviv, 6997801, Israel; 2 MicroDish BVH. Kruyt building, Padualaan 8, 3584 CH, Utrecht, The Netherlands; 3 Department of Plant Pathology and Microbiology The Robert H. Smith Faculty of Agriculture, Food and Environment, The Hebrew University of JerusalemRehovot, 7610001, Israel; 4 Department of Molecular Genetics, Weizmann Institute of ScienceRehovot, 7610001, Israel; 5 The Sackler School of Physics and Astronomy, Tel Aviv UniversityTel Aviv, 6997801, Israel; 6 Tauber Bioinformatics Research Center, University of HaifaHaifa, 3190501, Israel; 7 Center for Theoretical Biological Physics, Rice UniversityHouston, TX, 77005-1827, USA

## Abstract

Bacteria often use sophisticated cooperative behaviours, such as the development of complex colonies, elaborate biofilms and advanced dispersal strategies, to cope with the harsh and variable conditions of natural habitats, including the presence of antibiotics. *Paenibacillus vortex* uses swarming motility and cell-to-cell communication to form complex, structured colonies. The modular organization of *P. vortex* colony has been found to facilitate its dispersal on agar surfaces. The current study reveals that the complex structure of the colony is generated by the coexistence and transition between two morphotypes – ‘*builders’* and ‘*explorers’* – with distinct functions in colony formation. Here, we focused on the *explorers*, which are highly motile and spearhead colonial expansion. *Explorers* are characterized by high expression levels of flagellar genes, such as *flagellin* (*hag*), *motA, fliI, flgK* and *sigD*, hyperflagellation, decrease in ATP (adenosine-5′-triphosphate) levels, and increased resistance to antibiotics. Their tolerance to many antibiotics gives them the advantage of translocation through antibiotics-containing areas. This work gives new insights on the importance of cell differentiation and task distribution in colony morphogenesis and adaptation to antibiotics.

## Introduction

Bacterial swarming is a rapid and coordinated migration of bacteria across surfaces (Harshey, 2003; Kearns, 2010). Powered by flagella and often facilitated by surfactant secretions, the swarm generally contains correlated moving cells that stream in multiple directions, forming the classical long-lived patterns of whirls and jets (Darnton *et al*., 2010; Zhang *et al*., 2010). The soil bacteria *Paenibacillus vortex* is an example of a peritrichously flagellated species that exhibits a different type of swarming pattern while spreading on hard agar surfaces (1.5–2.25% w/v). *P. vortex* generates leading groups of cells named vortices that cooperatively whirl for long times (order of hours) around a common centre. The vortices expand in size and move outward from the inoculation point, leaving behind a trail of cells, resulting in a tendril-like colonial structure (Cohen *et al*., 1996; Ben-Jacob, 1997; 2003; Ben-Jacob and Cohen, 1997; Ben-Jacob *et al*., 1997; 1998). The vortices serve as building blocks for new colonies.

*P. vortex* is capable of collectively crossing regions containing a large variety of antibiotics by using ‘pioneering, swarming masses’ (Ingham *et al*., 2011). After reaching an antibiotic-free area, the pioneering bacteria regain their sensitivity to the same antibiotic (Ben-Jacob *et al*., 2000; Ben-Jacob, 2003; Ben-Jacob *et al*., 2004), suggesting temporary phenotypic adaptation. Adaptability to antibiotics and to changing environments can be achieved in colonies with task distribution and cell differentiation. Similar phenomena are found in persister cells in *Escherichia coli* (Balaban *et al*., 2004; Lewis, 2010), phenotypic switching between motile rods and robust cocci in *Paenibacillus dendritiformis* (Be’er *et al*., 2011), and the formation of biofilms in *Bacillus subtilis* (Stewart and Costerton, 2001). However, for *P. vortex*, the mechanism is unclear.

Here, we show that *P. vortex* colonies are composed of two coexisting morphotypes [bacterial phenotypes, display characteristics of distinct colonial morphologies (Ben-Jacob *et al*., 1998)], referred as ‘*builders*’ and ‘*explorers*’. Under normal growth conditions, the *builders* constitute the majority of the population; they are recognized by a relatively high growth rate in liquid and reduced swarming capabilities on solid medium. The *explorers*, on the other hand, have a reduced growth rate in liquid and are hyperflagellated, with elevated swarming capabilities on hard surfaces. The population is, thus, maintained by fast reproduction and expansion rates. *Explorers* are in the minority during liquid growth and are mainly found in the leading vortices on solid surface; however, they are extremely resistant to antibiotics, thus maintaining the group under antibiotic stress. The coexistence of these two subpopulations provides a unique advantage for colony growth. Our findings may have a general relevance to bacteria living in challenging and changing environmental niches, such as soil and rhizosphere.

## Results

### Isolation of *explorers* and *builders*

**Figure 1:**
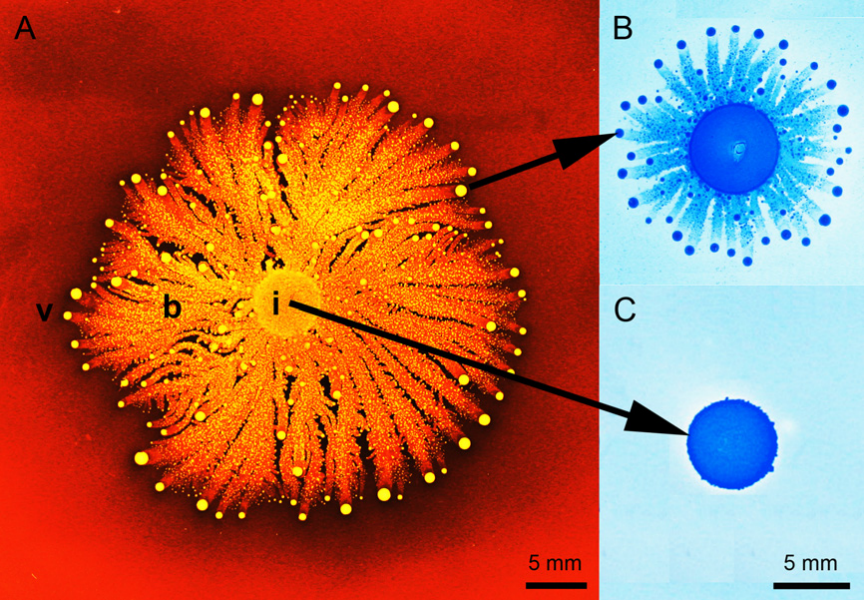
Transfer plating from different locations of the colony result in different patterns. A. *P. vortex*’s colony, grown on peptone agar (20 g l^−1^ peptone, 2.25% w/v agar) for 96 h at 30°C. The colours were inverted to emphasize higher cell densities using the brighter shades of yellow. v, vortices; b, branches; i, inoculation zone. B. Demonstrating replica plating from the vortices, grown on peptone for 48 h at 30°C. The colony is characterized by accelerated expansion rate (∼ 0.098 mm h^−1^) in comparison to mixed colony expansion rate (∼ 0.0625 mm h^−1^) (Fig. S1). C. Replica plating from inoculation zone, grown on peptone for 48 h at 30°C. The colony has shown no expansion as opposed to isolations from vortices.

A colony of *P. vortex*, spreading from the inoculation point on a hard agar, has the following structures: vortices at the leading edges, branches behind them and an inoculation zone at the centre (Fig. 1A). It has been shown that colonies initiated from bacteria taken from different structures of the colony generate distinct patterns (Ben-Jacob *et al*., 2004; Ben-Jacob and Levine, 2006). Following these observations, we isolated cells from two different structures: the vortices and the centre. The resulting colonies had distinct colonial morphologies with enhanced (1.5-fold) and no expansion rates, respectively (Fig. 1B and C), compared with normal expansion rate on peptone plates (Fig. S1A and B).

To test whether distinct colonial morphologies resulted from different functional properties of different morphotypes, we sorted and separated the population based on hypothetical tasks distribution of the bacteria in the colony.

#### Isolation of *explorers*

**Figure 2:**
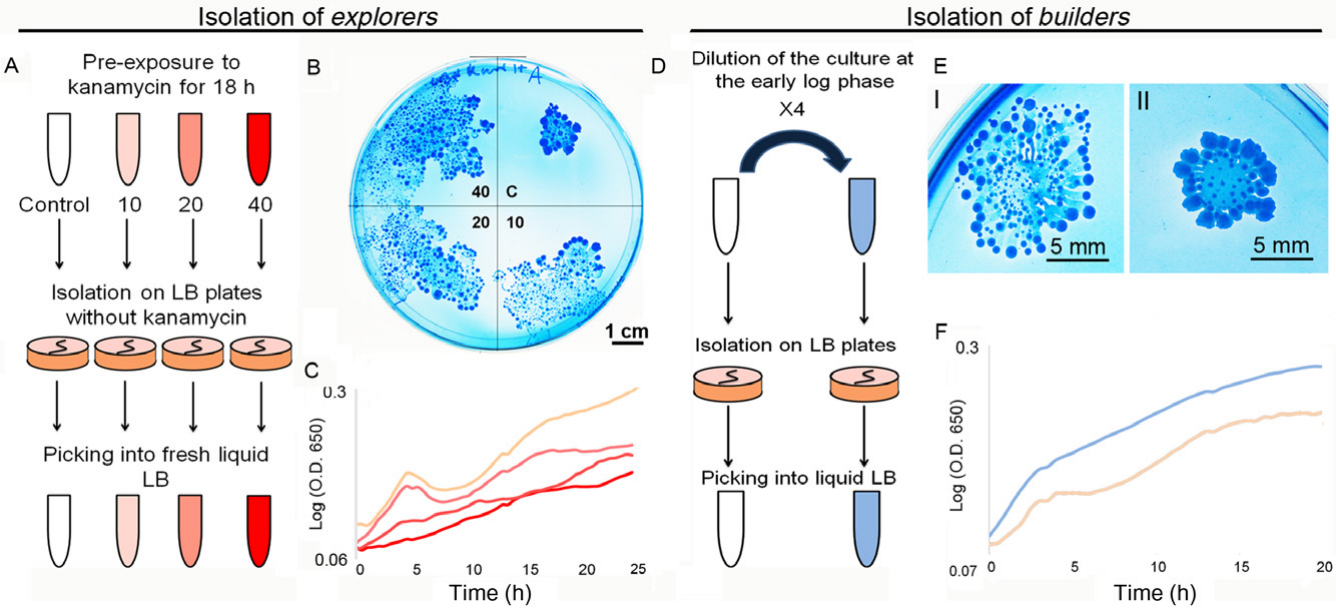
Isolation of *explorers* and *builders*.A. A scheme describing *explorers*’ isolation. *P. vortex* culture was pre-exposed to 10, 20 and 40 μg ml^−1^ kanamycin (represented by light pink, pink and red colours, respectively) for 18 h 37°C, and spread over LB plates without kanamycin (to measure spreading abilities). Cells were picked from microcolonies and inoculated in liquid LB without kanamycin (to evaluate growth rate and yield).B. *Explorers* have an increased colony diameter in comparison to the mixed culture upon growth on 1.5% (w/v agar) LB plates without antibiotics. Colony expansion diameter has increased with correspondence to kanamycin concentration. C –mixed culture, 10, 20 and 40 are μg ml^−1^ kanamycin respectively. Pictures were taken 24 h post-inoculation.C. Reduction in the growth rate of *explorers* isolated from different concentrations of kanamycin. All pre-exposed cultures show a reduction in the growth rate at the early (1–5 h) (*P* < 0.001) and mid (10–15 h) (*P* < 0.05) log phases, and growth yield after 20 h (*P* < 0.05) in liquid which is correlated to increase in kanamycin concentrations. The growth curves were made in kanamycin-free liquid LB medium. 10, 20 and 40 μg ml^−1^ kanamycin represented by light pink, pink and red colours respectively; cream colour, mixed culture.D. A scheme describing *builders*’ isolation. LB liquid culture was diluted at the early log phase (4 h) ×4 times to reduce the slow-growing *explorers* morphotype, then spread on LB agar. Blue colour, *builders*; white colour, non-treated culture.E. *Builders* picked from microcolonies were immediately diluted in liquid LB then inoculated on LB agar. I – The mixed culture shows normal expansion on solid medium. II – isolated *builders* show no expansion on solid medium. Pictures were taken 48 h post-inoculation.F. Growth curve was measured by inoculation of bacteria picked from microcolonies into a fresh LB medium for 20 h. *Builders* have shown increase in growth rate (compared with mixed cultures) at the early (1–4 h) (*P* < 1 × 10^−4^) and mid (9–13 h) (*P* < 0.01) log phases, and growth yield after 20 h (*P* < 1 × 10^−4^) in liquid.

Since vortices are located at the leading edge of the colony, we assumed that a vortex’s population might be more tolerant to different stresses, such as antibiotics. To test this hypothesis, *P. vortex* cells were treated with kanamycin (10, 20 and 40 μg ml^−1^) for 18 h. Pre-exposed cells were spread over antibiotic-free Luria–Bertani (LB) agar, and microcolonies were picked to measure the expansion diameter on agar, and the growth rate and the yield in liquid (20 h) (Fig. 2A). With the increase in kanamycin concentration (10, 20 and 40 μg ml^−1^), there was a decrease in both the growth rate (1.2-, 2.2-, 6.4-fold, respectively) and the growth yield in LB liquid (1.6-, 1.7-, 2-fold, respectively), in comparison to a mixed culture (untreated culture, composed of all morphotypes) (Fig. 2C). However, the expansion diameter of the resulting colonies surprisingly increased with the additions of kanamycin (more than 1.4-fold) (Figs 2B and S1). Since this population tended to spread fast and seemed to be found mainly at the leading edge of the swarming colony, we referred this morphotype as *explorers*.

#### Isolation of *builders*

Searching for additional subpopulations that may comprise *P. vortex* colonies, we exploited the *explorers*’ slow growth rate (Fig. 2C) and used repeated dilutions growth cycles to select against this phenotype (as described in Keren *et al*., 2004 for the elimination of persisters). The culture was grown in LB liquid O.N, diluted 1:100 into fresh LB and grown for 4 h, then diluted again as above for three more times (Fig. 2D). After each dilution, the culture resistance was challenged immediately with 20 μg ml^−1^ kanamycin, followed by a viable count on LB agar. After the fourth dilution, no growth was detected following exposure to kanamycin, indicating that kanamycin-resistant bacteria were no longer present in the culture, or at least extremely rare (data not shown). After four dilutions, the bacteria were spread over LB agar, and microcolonies were picked (immediately after their appearance ∼ 10 h) to measure the expansion diameter, as well as the growth rate and yield in liquid (20 h). The resulting lineage developed colonies with reduced expansion diameter (1.5-fold) on LB plates (Fig. 2E I and II), and elevated growth yield (1.5-fold) and growth rate (1.75-fold) in liquid LB (Fig. 2F). Testing the response to antibiotics, we found that the cells enriched by four dilution cycles were less (in comparison with the *explorers*) resistant to kanamycin both in liquid [minimal inhibitory concentration (MIC) of *builders* < 7.8 μg ml^−1^, *explorers* < 62.5 μg ml^−1^] and solid LB (Fig. S2). This morphotype was called *builders* as they seemed to be most common in the interior and appear to form the most common morphotype in the mature colony.

Since changes in growth rate and growth yield (20 h) in liquid medium and expansion diameter on solid surface were the most distinguishable characteristics in both *explorers* and *builders*, for further characterization and detection of the morphotypes, we have used these parameters (Fig. S3, Table S1). Cells that have presented an increase of ≥ 10% in expansion diameter and a decrease of ≥ 10% in the absorbance after 20 h in comparison to mixed culture were defined as *explorers* (Fig. S3, Table S1), while cells that exhibited the opposite phenotypes (the remainder) were defined as *builders*.

### The subpopulation of *explorers* is enriched in culture with antibiotics

**Figure 3:**
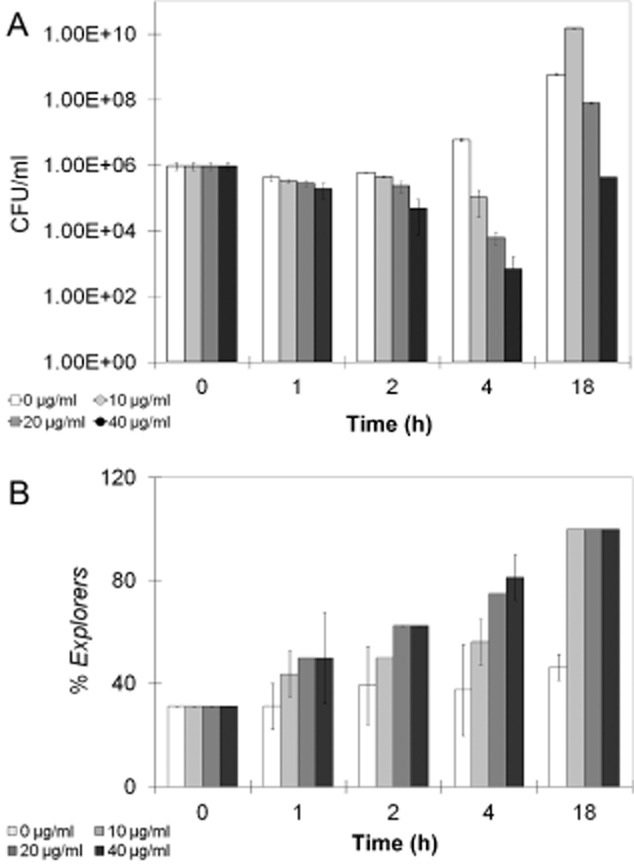
*Explorers* are constantly present in the culture. *P. vortex* was grown in the presence of 10, 20 and 40 μg ml^−1^ kanamycin for 18 h. At each time point, the total number of bacteria in the culture (CFU/ml) was measured, and *explorers*’ percentage in the culture was evaluated.A. CFU/ml was measured by culture dilution, plating and CFU counting at each time point. The number of bacteria under exposure to kanamycin decreases over time.B. Percentage of *explorers* in the culture was evaluated with both swarming assay on hard medium and absorbance assay at 650 nm at t = 20 h (as described in the *Isolation of explorers* paragraph and in Figs 2 and S3). The percentage of *explorers* in the culture increases both with time and with the increase in kanamycin concentration.

To test if *explorers* were already present in liquid culture of *P. vortex* and being enriched during exposure to kanamycin, the bacteria were grown in liquid medium and exposed to kanamycin (10, 20 and 40 μg ml^−1^). At different time points, two parameters were measured: total number of bacteria in the culture (Fig. 3A) and percentage of cells that scored as *explorers* (Fig. 3B). The total number of bacteria at each time point was determined by microcolonies counting (CFU/ml). Then, 8–16 microcolonies were picked for evaluation of *explorers*’ percentage. At t = 0, *explorers* comprised one third of the culture (Fig. 3B). As the population proliferated in the presence of kanamycin, the fraction of *explorers* appeared to increase and appeared to constitute > 99% of the culture after 18 h. During the first 4 h of culture in the presence of kanamycin, the total number of viable bacteria decreased (presumably, being killed or inhibited by the antibiotic), while the percentage of *explorers* increased, from 31% at t = 0 reaching 56% (10 μg ml^−1^), 75% (20 μg ml^−1^) and 81% (40 μg ml^−1^) at t = 4 h (Fig. 3A and B). Thus, the *explorers* are constantly present as a significant minority of *P. vortex*’s population, being enriched in the presence of kanamycin.

### The morphotypes stability

**Figure 4:**
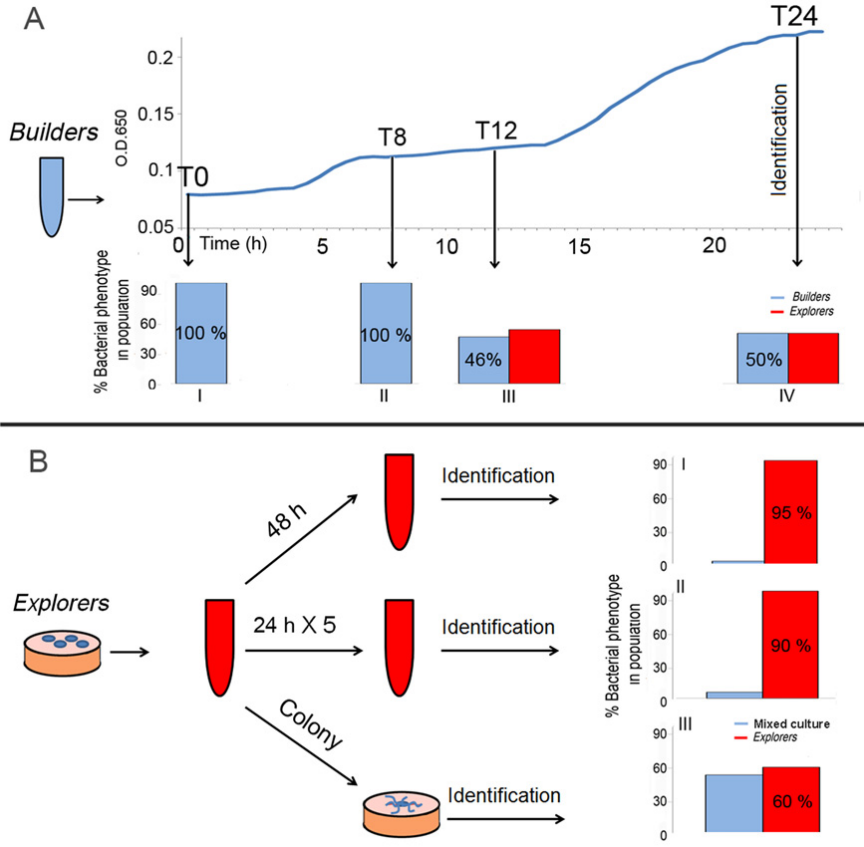
Morphotypes stability.A. Reversion of *builders* into the mixed culture. *Builders*’ enriched culture, obtained after × 4 transfers at the early log phase, was grown in liquid LB for 8 h, 12 h and 24 h. At each time point, samples were taken and *builders*’ percentage in the culture was evaluated. *Builders* are stable for at least 8 h (I,II). At 12 h, they revert back to the mixed culture (III), remaining ∼ 50% of the culture.B. Shows the stability of *explorers*’ morphotype after different treatments. *Explorers*’ microcolonies were inoculated in liquid LB O.N. (I) Samples were re-inoculated in liquid LB for 48 h; (II) Samples were re-inoculated in liquid LB for 24 h, five times continuously; (III) samples were inoculated as a colony on LB agar. After each treatment, *explorers*’ percentage in the culture was evaluated. The first two treatments (I and II) resulted in over 90% *explorers* in the culture. Only plating on hard medium induced the reversion to the mixed culture composition.

We have noticed that *builders*’ culture, obtained formerly by repeated dilutions at the early log phase, was unstable and changed within a few hours into a mixed culture. To test *builders*’ stability, we evaluated the time it takes for a population of *builders* to return into a mixed culture. To do so, purified *builders* were allowed to grow in liquid medium for 24 h and were sampled at different time points. The percentages of *builders* and *explorers* were evaluated using identification parameters presented at the first section. *Builders* were the only morphotype detected in the culture 8 h post-inoculation (Fig. 4A II). However, after 12 h, *builders* composed only of 50% of the culture (similar to mixed culture) (Fig. 4A III). After 24 h of incubation, the percentage of *builders* remained the same (Fig. 4A IV). *Builders* plated on LB agar reverted back to mixed colony within 24 h (data not shown). Cells isolated from each time point and exposed to kanamycin showed no growth after 8 h, and showed similar growth rate in comparison to mixed culture after more than 12 h (Fig. S4). Thus, *builders*’ culture remains stable for at least 8 h until *explorers* reappear within ≥ 12 h in liquid and solid media respectively.

One explanation of *explorers*’ appearance in the purified *builders*’ culture may be due to a rapid division of a few remaining *explorers*. To calculate the mean generation time of *explorers* that might have been left in the culture after dilution, we used the following equations:


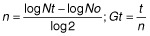


The number of divisions (*n*) was determined by dividing the log of the number of cells (*N_t_*) at time *t* minus the log of the number of cells at time zero (*N_0_*), by log2. The mean generation time (*G_t_*) was calculated by dividing time (*t*) by the number of divisions (*n*).

Under normal growth conditions, the mean generation time (*G_t_*) of a *P. vortex* culture (over 12 h) is approximately 2 h. However, according to estimation, the *G_t_* needed for a hypothetical fraction of 0.1% (1 × 10^3^ CFU/ml) *explorers* to reach 50% of the culture (1.5 × 10^8^ CFU/ml) in 12 h is 42 min; 1% *explorers* will reach the same concentration in 52 min. Thus, the estimated *G_t_* is much shorter than the observed *G_t_*. This indicates that the *explorers* probably emerged by a phenotypic transition rather than by replication.

The stability of *explorers* was tested by several approaches: First, isolated *explorers* were grown to high density (48 h) in liquid medium without kanamycin. The percentage of *explorers* in the resulting culture was 95% (Fig. 4B I). Second, isolated *explorers* were grown in liquid culture for 24 h, re-inoculated for another 24 h growth. After five such transfers, the resulting culture contained 90% *explorers* (Fig. 4B II). Finally, isolated *explorers* were inoculated on a fresh LB agar. The resulting colony (24 h growth) contained 60% *explorers* only (Fig. 4B III). The *explorers* morphotype was shown to be extremely stable in liquid, meaning almost no reversion to the mixed culture occurred. However, when *explorers* were inoculated on solid surface and formed a colony, only 60% *explorers* remained, that is the transition to mixed culture has occurred showing typical growth rate and swarming capabilities (not shown). We have shown that both morphotypes can develop into the other; their characteristics are stable. However, the stability of these morphotypes is different. We further focused on *explorers* because of their stability and importance of phenotypic resistance to antibiotics.

### *Explorers* are hyperflagellated

**Figure 5:**
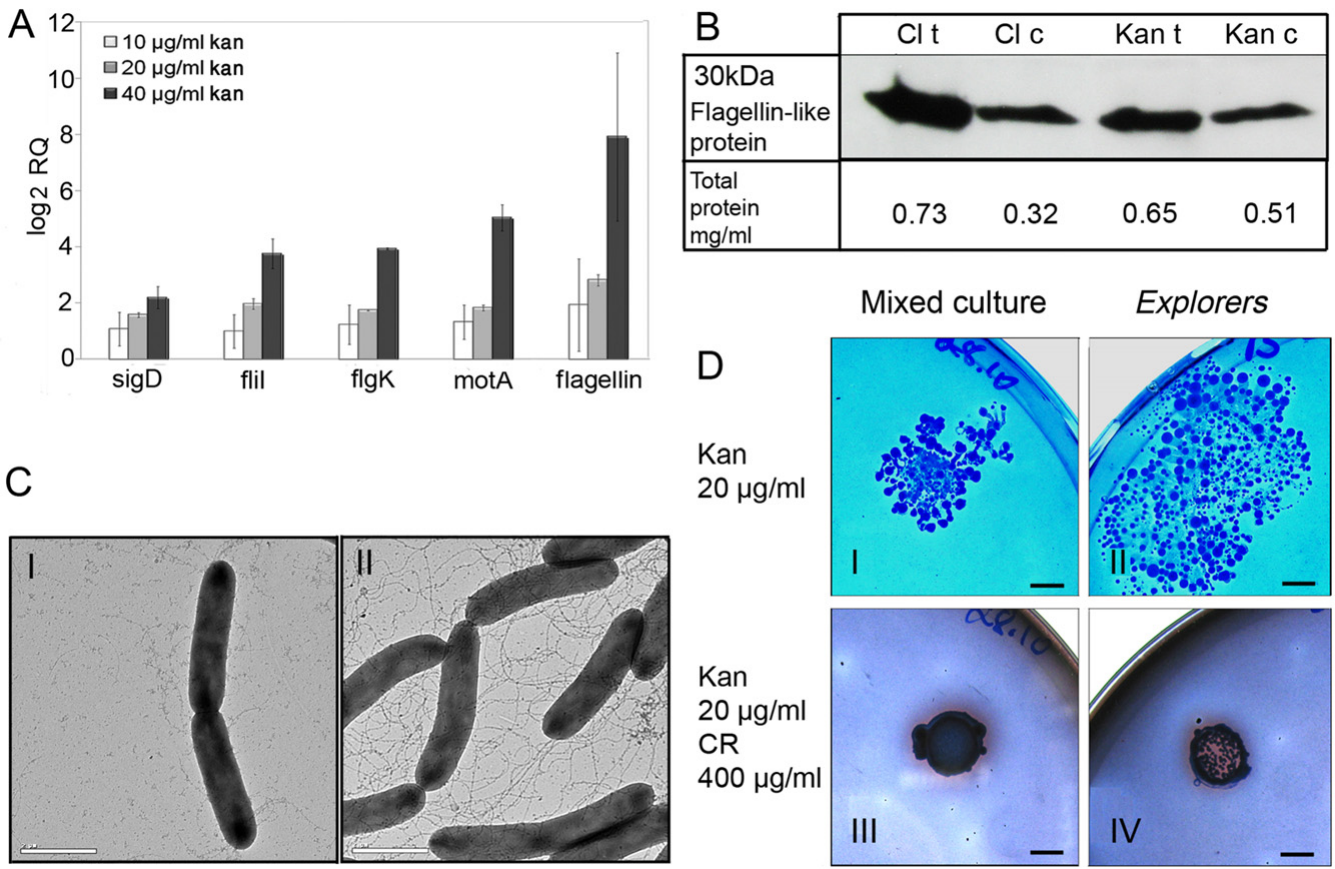
*Explorers*’ are hyperflagellated.A. Real-time PCR. Increase of flagella gene expression is in correlation to kanamycin concentrations. *Explorers* were isolated after pre-exposure to 10 (white), 20 (light grey) and 40 μg ml^−1^ (dark grey) kanamycin for 18 h, and examined relatively to the mixed culture. *Y*-axis represents the binary logarithm of genes relative expression (ΔΔCt). *X*-axis: *sigD, fliI, flgK, motA* and *flagellin* (*hag*).B. Western blot with anti-flagellin antibody. Elevation of flagellin in *explorers* isolated from either 40 μg ml^−1^ kanamycin (Kan t) or 15.6 μg ml^−1^ – chloramphenicol (Cl t). Non-treated bacteria (Cl c, Kan c). The number of cells in all samples (10^10^ CFU/ml) was equalized; thus, the levels of proteins presented here are per cell.C. TEM imaging. Increase of flagella filaments in *explorers* in comparison to *builders*. Scale bar = 2 μm.D. In the presence of Congo red, flagella production is inhibited. Mixed culture *and explorers* were plated on agar plates with 20 μg ml^−1^ kanamycin (I and II) and 20 μg ml^−1^ kanamycin + 400 μg ml^−1^ Congo red (III and IV) and incubated for 24 h. Absence of flagella in the presence of Congo red prevented swarming of the mixed as well as *explorers* colonies. Scale bar = 5 mm.

To test if the enhanced swarming of the *explorers* is supported by hyperflagellation, we examined the level of flagella synthesis. First, we tested the expression of flagellin and motility-related genes by real-time PCR (polymerase chain reaction) using primers amplifying the following genes: *flagellin* (*hag*)*, flgK, fliI, motA* and *sigD* (hypothetical annotations of flagella genes presented in Table S4). These genes were selected based on the outcome of the Genome Holography bioinformatics analysis of microarray chip results (Madi *et al*., 2008; Roth *et al*., 2011). For more details, see Figs S5 and S6, and Table S2. The analysis was done to provide a functional representation of flagella biosynthesis genes. Comparing the expression values between mixed culture and isolated *explorers*, we found that the *explorers* exhibit more than a 1.5-fold elevation in the expression of flagella and motility-related genes (Fig. 5A). We also found that the expression levels increased with kanamycin concentration (pre-exposure to 10–40 μg ml^−1^ kanamycin) (Fig. 5A).

Second, flagellin levels were quantified in *explorers* and mixed cultures by Western blot. Two types of antibiotics were used for these experiments to verify if the phenomenon was unique to kanamycin or more general. Quantitative Western blot was performed on extraction of flagella proteins from equal CFU of *explorers*, isolated from either 40 μg ml^−1^ kanamycin or 15.6 μg ml^−1^ chloramphenicol for 18 h, and mixed cultures using anti-flagellin antibody. ImageJ software (National Institutes of Health, Bethesda, MD, USA) and total protein analyses have shown elevation of approximately 2.2-fold and more than 1.2-fold in flagellin extracted from *explorers* isolated from chloramphenicol and kanamycin, respectively, in comparison to mixed culture (Fig. 5B).

Phenotypic flagella testing by transmission electron microscope (TEM) imaging using *explorers* samples isolated from the same antibiotics as in previous paragraph has shown similar results. *Explorers* were found to have significantly more flagella compared with mixed culture (Fig. S7). Finally, TEM images of *explorers* and *builders* taken from microcolonies grown for few hours on LB plates have shown elevated number of flagella in *explorers* in comparison to *builders* (Fig. 5C). These results confirm that *explorers* are hyperflagellated.

However, it has been previously suggested that *P. vortex* may have additional ways to move on solid surface (i.e. with the aid of pili) (Sirota-Madi *et al*., 2010). To test whether flagella are essential for colony expansion on solid medium, we have grown *explorers* on plates with 400 μg ml^−1^ Congo red (known to inhibit flagella production) (Arnold and Shimkets, 1988; Velicer *et al*., 1998) with and without 20 μg ml^−1^ kanamycin. Congo red has previously been shown to inhibit flagellar motility of *P. vortex* at this concentration (Ingham and Ben-Jacob, 2008). In a separate experiment, we have shown that incubation of the *explorers* in the presence of 400 μg ml^−1^ Congo red did not affect the growth rate of the bacteria with and without pretreatment with kanamycin (data not shown). We found that, on plates containing Congo red, the expansion of both colonies (mixed culture and *explorers*) was inhibited (Fig. 5D III and IV). These results suggest that flagella are essential for *explorers*’ expansion on solid surface. The combination of all the results approves that enhanced swarming of the *explorers* is supported by hyperflagellation.

### Kanamycin concentration is negatively correlated with cellular ATP levels in *explorers*

**Figure 6:**
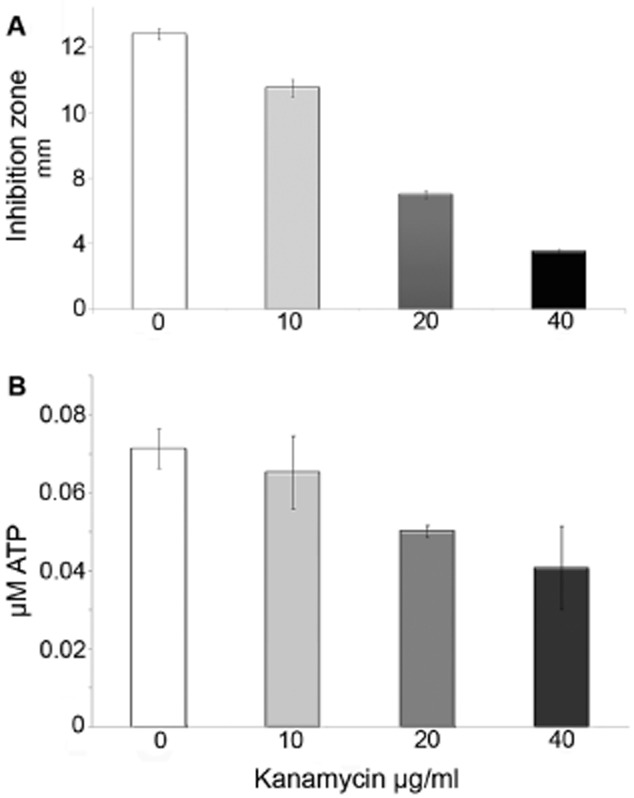
Resistance to kanamycin is negatively correlated with ATP levels. *Explorers* were isolated from 10, 20, 40 μg ml^−1^ kanamycin (*x*-axis).A. The resistance of *explorers* to kanamycin was examined using disk diffusion assay containing 50 μg kanamycin. The bars represent the averaged radius of inhibition zone, which indicates the resistance level of *explorers* (*P* < 1 × 10^−4^) (i.e. smaller radius indicates higher resistance). The resistance increases with the increase in kanamycin concentrations during the pre-exposure.B. The bars show the level of cellular ATP of *explorers* isolated from similar concentrations of kanamycin (*P* < 0.05). The assay was performed using ATPlite kit. There is a negative correlation between ATP cellular levels and kanamycin concentrations. Combining linear dependences of A and B, one can see that the cellular levels of *explorers*’ ATP are negatively correlated with resistance to kanamycin.

To test the resistance of *explorers* to kanamycin, we used disk diffusion assay. *Explorers*, isolated from different concentrations of kanamycin (10, 20, 40 μg ml^−1^), were plated on LB agar. The resistance of *explorers* to kanamycin was indicated by inhibition zone caused by a disk containing 50 μg kanamycin placed at the centre of the plate (Fig. 6A). We found that *explorers* isolated from higher concentrations of kanamycin have shown an increased resistance (Fig. 6A).

It has been previously shown that resistance to aminoglycosides (kanamycin), accompanied with reduced growth rate in liquid, is often associated with a reduction in membrane potential (proton motive force, PMF) and ATP levels due to defects in the electron transport chain (Wilson and Sanders, 1976; Proctor *et al*., 1998; Melter and Radojevic, 2010). Motivated by this connection, we tested ATP levels in the mixed and *explorers* cultures to see if there is dependence between cellular ATP and resistance to kanamycin. We found a decrease of approximately 10%, 30% and 45% in cellular ATP levels for the *explorers* isolated from 10, 20 and 40 μg ml^−1^, respectively, in comparison to the mixed culture (Fig. 6B). Since the concentrations of kanamycin during the pre-exposure are positively correlated with *explorers*’ resistance (Fig. 6A) and negatively correlated with cellular ATP levels (Fig. 6B), one may conclude that the resistance to kanamycin is negatively correlated with ATP levels. These findings are in agreement with the resistance to kanamycin observed in bacteria with reduced cellular ATP (e.g. small colony variants, persisters), and with negative correlation found between growth rate and kanamycin concentration (Fig. 2C).

### The tolerance of *explorers* to other classes of antibiotics

**Figure 7:**
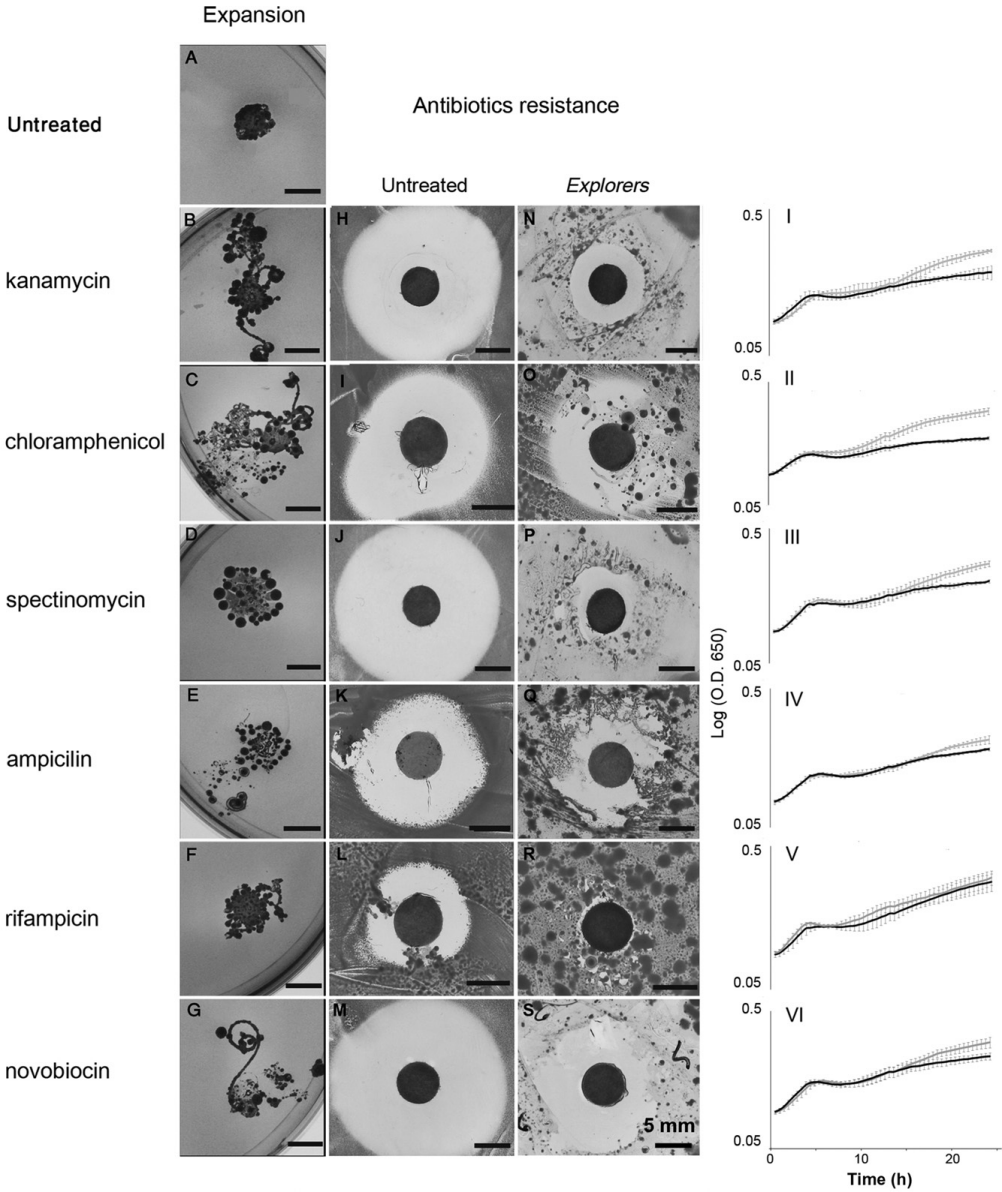
*Explorers* isolated from different types of antibiotics. The mixed culture was grown for 18 h in liquid LB with various antibiotics (indicated on the left) at MICs. The expansion diameter, resistance to antibiotics, growth rate and growth yield of pre-exposed (*explorers*) and non-exposed culture were measured.A–G. Expansion on LB agar. The pictures were taken after 24 h of growth on LB agar.A. Colonies of non-exposed culture.B–G. Colonies of *explorers* isolated from different types of antibiotics.H–S. Disk diffusion assay.H–M. Mixed cultures, N–S. *explorers*. Resistance to antibiotics was examined on LB plates using disk diffusion assay containing 50 μg kanamycin (H,N), 50 μg chloramphenicol (I,O), 5 μg spectinomycin (J,P), 5 μg ampicillin (K,Q), 0.1 μg rifampicin (L,R) and 5 μg novobiocin (M,S). The smaller the radius of inhibition zone, the higher the resistance level of the bacteria. We can see that *explorers* have a smaller inhibition zone, i.e. have a higher resistance. (I–VI) Growth curves of *explorers* (black) and mixed culture (grey). *Explorers* show a reduction in the growth rate at the mid (10–20 h) log phase (*P* < 0.002), and growth yield after 20 h (*P* < 0.008) in liquid in comparison to the mixed culture. Exceptional are the *explorers* isolated from ampicillin and rifampicin, (*P* < 0.03) and (*P* < 0.04), respectively, which shows similar growth kinetics to the mixed culture.

To test if the *explorers*’ resistance is restricted to kanamycin or is a general reaction to antibiotics, we further examined the tolerance of *explorers* to six other types of antibiotics that represent five distinct antibiotic families with different modes of action (kanamycin, spectinomycin, chloramphenicol, novobiocin, rifampicin and ampicillin; Table S5). *P. vortex* bacteria were exposed to the MIC of each antibiotic for 18 h. The expansion diameter on LB agar, growth rates and growth yield (20 h) of pre-exposed bacteria were examined. The results of pre-exposure to different types of antibiotics were similar to pre-exposure to kanamycin (isolation of *explorers*): wider expansion diameter on solid medium (Fig. 7A–G), and decreased growth rates and growth yields after 20 h of bacteria in liquid medium (Fig. 7I–VI). These findings may indicate that *explorers*’ responses to antibiotic stress are not unique to kanamycin but also applies to a wide range of structurally unrelated antibiotics.

In another set of experiments, we tested the tolerance of *explorers* to different types of antibiotics incorporated in agar. *Explorers* isolated from different types of antibiotics were inoculated on LB plates and examined using disk diffusion assays. The disks used in each assay contained the same antibiotics as the former pre-exposure. In all cases, *explorers* were tolerant to the same antibiotics during secondary exposure, having a smaller radius of inhibition than the control (Fig. 7H–S).

## Discussion

This study reveals a novel aspect in the morphogenesis of bacterial colonies and adaptation to antibiotics. Since *P. vortex* cannot yet be studied using genetic means (it is not currently transformable), we have used other methods to distinguish and investigate *P. vortex*’s subpopulations. We have shown that *P. vortex* cultures can contain at least two coexisting subpopulations (‘*builders’* and ‘*explorers’*) with different task distribution that together form a unique colony pattern. The expansion patterns of cells isolated from different locations of *P. vortex* colony (centre and vortex) (Fig. 1) implies that *builders* comprise mainly the internal parts of the colony, while *explorers* are located mostly in the periphery of the colony. The existence of *explorers* and *builders* at distinct locations of the expending colony is consistent with the model explaining swarming migration involving elongation of dendrites in *B. subtilis*. When swarmers accumulate at the tip of the dendrite establishing a leader track and non-swarmers that group at the edge of the inoculation zone build the dendrite by growth and multiplication (Matsuyama and Matsuyama, 1993; Hamze *et al*., 2011).

The phenomenon of two distinguishable cell types that coexist within the clonal population is sometimes referred to as ‘bistability’. Bistability is exemplified in *E. coli* by persister cells, and in *B. subtilis* by genetic competence, spore formation/cannibalism and swimming/chaining. Bistability might be advantageous, allowing cells to hedge their bets so that a few cells enter a state that would be better adapted to one circumstance or another should that circumstance arise (Dubnau and Losick, 2006). The coexistence of *explorer* and *builder* morphotypes as subpopulations suggests that their transition is also bistable, although we do not exclude further morphotypes. The proportion of the two morphotypes in the culture can be changed, and both morphotypes can revert in response to specific conditions, creating a mixed culture that is apparently necessary for proper colony formation. However, in contrast to ‘classical’ bistability, the stability, especially of *explorers*, appears to be very high (the transition rate is more than 24 h × 5 transfers).

In the soil, bacteria often cope with many fluctuations in the environment. This may be particularly true for swarming (‘travelling’) bacteria, such as passage from watery to hard surfaces. The coexistence of *explorers* and *builders* in a planktonic population may be essential for a rapid translocation and colonization in changing environment. One such example would be the ability of *explorers* to swarm through areas containing high levels of antibiotics, moving the colony through stressful niches, whereas the ability of *P. vortex* to revert to the mixed culture composition allows to recreate the colony in a newly colonized area.

The appearance of the different phenotypes of *explorers* when exposed to different concentrations of kanamycin may suggest a direct effect of kanamycin rather than intrinsic characteristics of *explorers*. The effect of non-lethal levels of antibiotics on bacterial motility has been previously reported (Kawamura-Sato *et al*., 2000; Park *et al*., 2008; Shen *et al*., 2008). However, this effect is usually limited to the exposure period and disappears after the removal of the antibiotics. The fact that the *explorers*’ phenotype is extremely stable may suggest that the *explorers*’ emergence is attributed to selection and enrichment by kanamycin. Nevertheless, this result does not disprove an additional effect of kanamycin on flagella genes.

The time it takes for bacteria to initiate swarming is called ‘swarm lag’ (Kearns, 2010). The increase in the number of flagella per cell in *explorers* may shorten the swarm lag in several ways: Hyperflagellated bacteria are prepared to swarm sooner after inoculation (reduced swarm lag) (Kearns and Losick, 2005; Kearns, 2010). The high number of flagella entangled with neighbouring cells may reduce the critical density of cells that is necessary to form raft that serves for cell translocation (Kirov *et al*., 2002; Ingham and Ben-Jacob, 2008; Kearns, 2010). This hypothesis may explain the previously observed phenomena of a short swarm lag in *P. vortex* (Ingham *et al*., 2011) and suggest that hyperflagellation can be an advantage for colony formation.

In this work, we have found that the fast-spreading *explorers* are hyperflagellated on one hand, and have reduced levels of cellular ATP on the other. It was previously shown that bacteria utilize PMF for flagella biosynthesis and motor function (Sowa and Berry, 2008; Minamino *et al*., 2011). Additionally, the flagellar switch–motor complex was found to consume ATP (Zarbiv *et al*., 2012). Hence, a possible explanation of ATP reduction could be in higher consumption of PMF and ATP by flagella biosynthesis and motor function, possibly as part of a diversion of a higher than normal fraction of the cells energy budget towards motility. The negative correlation between the expression levels of flagella genes and ATP levels in *explorers* supports this idea.

Reduction in ATP levels and a lower growth rate in liquid culture may also explain the increased resistance to kanamycin. There is some evidence that differentiation into swarmers lowers metabolic activity unrelated to motility, and changes the physiology of the cell (Armitage, 1981; Kim and Surette, 2004). *Explorers* may be an example of swarmers that owe their adaptation to antibiotics to physiological changes (such as lower metabolic activity). This enhanced tolerance of *explorers* may explain the previously shown phenomenon of ‘pioneers’ and ‘secondary swarm fronts’ (highly tolerant subpopulations that cross antibiotic-containing areas) (Kim *et al*., 2003; Lai *et al*., 2009; Ingham *et al*., 2011). This phenomenon was attributed so far to temporary adaptive differentiation of the swarming cells into antibiotic-tolerant phenotype (such as persisters) (Kim *et al*., 2003; Lai *et al*., 2009).

Additional explanation for increased antibiotic resistance may be attributed to the high local cell density and mobility of the swarming colony (Butler *et al*., 2010). The highest cell density in a colony of *P. vortex* is usually at the centre and the vortices (tips of the colony, Fig. 1A). *Explorers*, which comprise mainly the vortices, are responsible for the mobility and create a high local cell density, thus may have a role in the adaptive tolerance of the colony against antibiotics.

## Experimental procedures

### Culture

Liquid culture of *P. vortex* was in LB broth at 37°C with vigorous shaking at 220 rpm. Growth in liquid culture was measured at OD_650_ using EL808, BioTek spectrophotometer (Winooski, VT, USA). For colony growth, peptone plates contained 20 g l^−1^ peptone, 2.25% w/v agar, were poured with 12 ml of medium, and were allowed to solidify and dry until 1 g of their original weight was lost. Peptone plates were incubated for 24–96 h at 30°C. For swarming assays and isolation of bacteria, LB (Difco) agar (1.5% w/v) at 37°C was used. Note that peptone plates were not used for swarming assay since *explorers* have a reduced growth rate, which challenges their expansion on minimal medium. Pre-exposure was performed with kanamycin, chloramphenicol, ampicillin, rifampicin, spectinomycin and novobiocin (Sigma) (Table S5). All antibiotics were dissolved in DDW (double-distilled water) or ethanol (chloramphenicol), filter-sterilized and added to O.N. LB liquid culture diluted 1:100 at the appropriate concentrations. For assays in microtiter plates, approximately 10^5^/ml cells were inoculated into 96 wells containing 100 μl LB with or without antibiotics in 37°C with shaking. Inoculations for swarming were made by depositing a 5 μl drop of an O.N. or pre-exposed culture grown in LB broth onto the centre of 1.5% w/v LB plate and incubated for 24 h at 37°C. For swarming inhibition, Congo red (Sigma) was dissolved in distilled water, filter-sterilized and added to LB or peptone agar just before pouring at a final concentration of 400 μg ml^−1^.

### Disk diffusion assay

10^5^ cells from liquid cultures were swabbed uniformly across the LB agar. The plates were left to dry for 10 min. A filter paper disk with 50 μg kanamycin, 5 μg ampicillin, 50 μg chloramphenicol, 5 μg spectinomycin, 5 μg novobiocin and 0.1 μg rifampicin was placed at the centre of the plate. Plates were incubated O.N. at 37°C, and zones of inhibition were measured.

### Image capture and light microscopy

Macroscopic images of the colonies were taken using digital camera (PowerShot A640, Canon, Melville, NY, USA). Colonies were stained with Brilliant Blue 0.1%, methanol 50% and acetic acid 10% to obtain higher contrast images. For microscopic images, pictures were taken using Olympus microscope under a total magnification of 200-fold (Olympus, Japan).

### TEM

Actively swarming *P. vortex* were gently placed on the TEM grid by simply placing the grid against the surface of 1.5% LB plates. The grid with the collected sample was stained with uranyl acetate (negative staining) and observed in JEM 1200 EX electron microscope (JEOL Ltd., Akishima, Tokyo, Japan).

### ATP measurements

*Explorers* isolated from 10, 20 and 40 μg ml^−1^ kanamycin were grown for 18 h in liquid medium. Bacteria from each culture were diluted to 10^6^ CFU/ml and treated according to manufacturer protocol (ATPlite, PerkinElmer, Waltham, MA, USA). The amount of emitted light (directly proportional to the ATP concentration) was measured with a luminometer. The ATP content (μM) of the cells was calculated using an ATP standard curve. ATP concentration was related to total cell extracted volume.

### Real-time PCR

*P. vortex* RNA was used for reverse transcription RT-PCR Thermo Scientific kit; real-time PCR was performed using Applied Biosystems SYBR Green reagent kit (Carlsbad, CA, USA). Flagella primers are described in Table S3: Amplification of the housekeeping gene 16srRNA with the primers (15,16 Table S3) was used as a control. Thermocycling programme included an initial denaturation step of 10 min at 95°C. Thermal cycling proceeded with 40 cycles of 95°C for 15 s and 60°C for 1 min. All amplifications and detections were carried out in StepOnePlus real-time PCR system. At each cycle, accumulation of PCR products was detected by monitoring the increase in fluorescence of the reporter dye from the dsDNA-binding SYBR Green. After the PCR, a dissociation curve (melting curve) was constructed in the range of 60°C–95°C. All data were analysed using the StepOne software v2.1. The real-time PCR amplification efficiency percentage of flagellin (hag), *sigD*, *motA*, *fliI, flgK* was 115.3%, 96.1%, 88.1%, 105.1%, 91.1% respectively. The *R*^2^ of all these standard curves was higher than 0.99.

### Purification of flagellin

Flagellin was extracted from *P. vortex* culture, grown O.N. in LB. Bacteria were centrifuged for 5 min at 2700 g, re-suspended in phosphate buffered saline and diluted to 10^10^ CFU/ml. Flagella were removed by sonication in Ultrasonic Cleaner (D80H, Taiwan) with heat treatment at 40°C for 10 min. Following sonication, bacteria were vigorously vortexed for 10 s and centrifuged at 20 800 *g* for 15 min. The supernatant containing flagellin was then collected and kept in 4°C.

### SDS-PAGE and Western blot

The presence of flagellin was confirmed by 30 kDa band on SDS-PAGE 4–20% gradient gel (Bio-Rad Laboratories, Hercules, CA, USA). In addition, Western blot was performed using 1% w/v dried skim milk for blocking, 1:5000 IgG-HRP secondary anti-rabbit antibody and 1:1000 polyclonal anti-flagellin. Polyclonal anti-flagellin was raised in rabbits against the synthetic peptides: 1. RINRAADDAAGLAISEKMR: 2. GAVQNRLEHTVNNLG. (Eurogentec, Belgium; http://www.eurogentec.com). For relative quantification of flagellin, ImageJ 1.46 software was used.

## Supporting Information

**Fig. S1.** Kinetics of colony development on peptone agar. *P. vortex* was grown for 18 h in liquid LB with and without 10 μg ml^-1^ kanamycin. Cells were then inoculated on peptone plates, (20 gl^-1^, 2.25% w/v agar) and grown in 30°C for 3 days (A). Pictures were taken after 24, 48 and 72 h. Scale bar = 5 mm. Colony diameter (branches width, mm), was measured after each time point (B). **Fig. S2**. *Builders*’ susceptibility to kanamycin. *Builders*’ enriched culture, *explorers* and mixed culture were examined for resistance to kanamycin using disk diffusion assay. The bars represent the averaged values of inhibition radius. A significant difference is found between *builders*’ and *explorers*’ inhibition radius with *P* < 8.6×10^-07^. Pictures on top of each bar represent the inhibition zone of each morphotype respectively. Scale bar – 5 mm. **Fig. S3.** Identification of *explorers*. Cells isolated from 10, 20 and 40 μg ml^-1^ kanamycin were spread over LB agar. Microcolonies were picked, re-inoculated on LB agar, and the expansion diameter was examined. An example of swarming assay of four isolated samples after 24 h of incubation on LB plates. The expansion diameter (mm) presented in Table S1A. **Fig. S4.** Growth of *builders*’ enriched culture in the presence of kanamycin. *Builders*’ enriched culture (blue) obtained after X 4 transfers at the early log phase, were grown in liquid LB for 8 h (A), 12 h (B) and 24 h (C). After each time point, samples were spread over LB agar, then microcolonies were immediately picked and added into liquid LB with (dashed line) and without (smooth line) 20 μg ml^-1^ kanamycin, and growth curves were examined. Mixed culture grown to the same time points were taken as a control (black). (A) After 8 h, the *builders* did not grow in the presence of kanamycin compared with mixed culture. (B and C), After 12 h and 24 h, the culture had the same growth kinetics as the mixed culture, suggesting *builders* are stable up to 8 h but revert to the mixed culture composition by 12 h. **Fig. S5.** Gene expression of *P. vortex* under kanamycin exposure. Gene expression profile of *P. vortex* culture after exposure to kanamycin. Normalized log signals (natural log, based on ‘e’) of ‘control’ and ‘kanamycin’ duplicates were averaged, and the difference between the averages was calculated. Upregulated genes are all the genes whose expression ratio is above 1.5 (log C > 0.4). The genes were filtered based on the presence of the kanamycin-induced upregulation and divided according to functional categories annotated by TIGR. The bars represent the percentage of upregulated genes from the total number of genes at each category. **Fig. S6.** Holographic network of *P. vortex* flagella genes. Holographic network of flagella genes after 18 h of exposure to antibiotics. The correlation lines connecting the genes were set by correlation threshold greater than 0.7 and smaller than -0.4. The matrices sorted by dendrogram algorithm. **Fig. S7.** Increase of flagella filaments number in *explorers* isolated from 40 μg ml^-1^ kanamycin (A,B) or 15.6 μg ml^-1^ chloramphenicol (C,D). A,C Mixed culture. B,D *explorers*. Magnification of X 50 K. Scale bar = 500 nm. **Fig. S8.** RAPD-PCR of *explorers*. Columns 1 – 100bp Marker (Tal Ron). Columns 2,5 – *Explorers*. Columns 3,6 – *B. subtilis* 168 (negative control). Columns 4,7 – *P. vortex* (mixed culture). Primers for amplification – 16Sr*RNA* (2–4), SPOVFA (5–7). Amplification profiles of *explorers* and *P. vortex* were similar under both primers in contrast to *B. subtilis* 168, indicating that *explorers* and *P. vortex* are the same species. **Table S1.** Detection of *explorers* in the culture. (A) Colony expansion diameter (mm) of isolated *explorers* (Fig. S3). (B) The absorbance at O.D._650_ of the same isolates, after 20 h of growth. Isolates that have shown 10% increase in colony diameter (A) and over 10% decrease in the absorbance after 20 h (B) were defined as *explorer*. The values of each isolate were compared with the average value of the control (bold). Isolates with a colony diameter value > 7.01 and O.D < 0.207 were identified as *explorers* (marked in grey). Evaluation of *explorers*’ percentage was determined by division of the number of samples identified as *explorers* by the total number of samples. **Table S2.** Expression of flagella and chemotaxis genes under exposure to kanamycin. All genes represented in the table have expression ratio above 1.5 (log C > 0.4). Genes’ IDs were represented by the Query Locus Tag, published in NCBI’s GEO [GSE35271]. **Table S3.** Primers for flagella genes, used for RT-PCR. *Genes’ IDs were represented by the Query Locus Tag, published in NCBI’s GEO [GSE35271]. **Table S4.** Annotation of hypothetical genes. **Table S5.** Emergence of *explorers* under different antibiotics.
