# Supplementary material for: Identification and characterization of a highly motile and antibiotic refractory subpopulation involved in the expansion of swarming colonies of Paenibacillus vortex
Source: Environ Microbiol. 2013 Jun 14;15(9):2532–44. doi: 10.1111/1462-2920.12160 (PMC3908376; doi:10.1111/1462-2920.12160)
Supplement: Supplementary file 1 — Fig. S1. Kinetics of colony development on peptone agar. P. vortex was grown for 18 h in liquid LB with and without 10 μg ml-1 kanamycin. Cells were then inoculated on peptone plates, (20 gl-1, 2.25% w/v agar) and grown in 30°C for 3 days (A). Pictures were taken after 24, 48 and 72 h. Scale bar = 5 mm. Colony diameter (branches width, mm), was measured after each time point (B). Fig. S2. Builders’ susceptibility to kanamycin. Builders’ enriched culture, explorers and mixed culture were examined for resistance to kanamycin using disk diffusion assay. The bars represent the averaged values of inhibition radius. A significant difference is found between builders’ and explorers’ inhibition radius with P < 8.6×10^-07^. Pictures on top of each bar represent the inhibition zone of each morphotype respectively. Scale bar – 5 mm. Fig. S3. Identification of explorers. Cells isolated from 10, 20 and 40 μg ml-1 kanamycin were spread over LB agar. Microcolonies were picked, re-inoculated on LB agar, and the expansion diameter was examined. An example of swarming assay of four isolated samples after 24 h of incubation on LB plates. The expansion diameter (mm) presented in Table S1A. Fig. S4. Growth of builders’ enriched culture in the presence of kanamycin. Builders’ enriched culture (blue) obtained after X 4 transfers at the early log phase, were grown in liquid LB for 8 h (A), 12 h (B) and 24 h (C). After each time point, samples were spread over LB agar, then microcolonies were immediately picked and added into liquid LB with (dashed line) and without (smooth line) 20 μg ml-1 kanamycin, and growth curves were examined. Mixed culture grown to the same time points were taken as a control (black). (A) After 8 h, the builders did not grow in the presence of kanamycin compared with mixed culture. (B and C), After 12 h and 24 h, the culture had the same growth kinetics as the mixed culture, suggesting builders are stable up to 8 h but revert to the mixed culture composition by 12 h. [file emi0015-2532-sd1.doc]

**Supplementary information**

**S1. *Explorers’* colony formation on peptone agar**

In order to examine the formation of *explorers’* colony on peptone agar, *P. vortex* pre-exposed to 10 µg/ml kanamycin were cultured on peptone plates (20 g/l peptone, 2.25% w/v agar) without antibiotics. The expansion rate of *explorers* was higher at the first 48 h (~0.0875 mm/h) in comparison to the expansion rate of mixed culture, (~0.0625 mm/h). After 48 h the expansion rates of *explorers* and mixed cultures become equal (~0.2 mm/h), however there were more vortices in *explorers’* colony, the structure was less organized and colony diameter was larger than the mixed culture (Fig. S1).


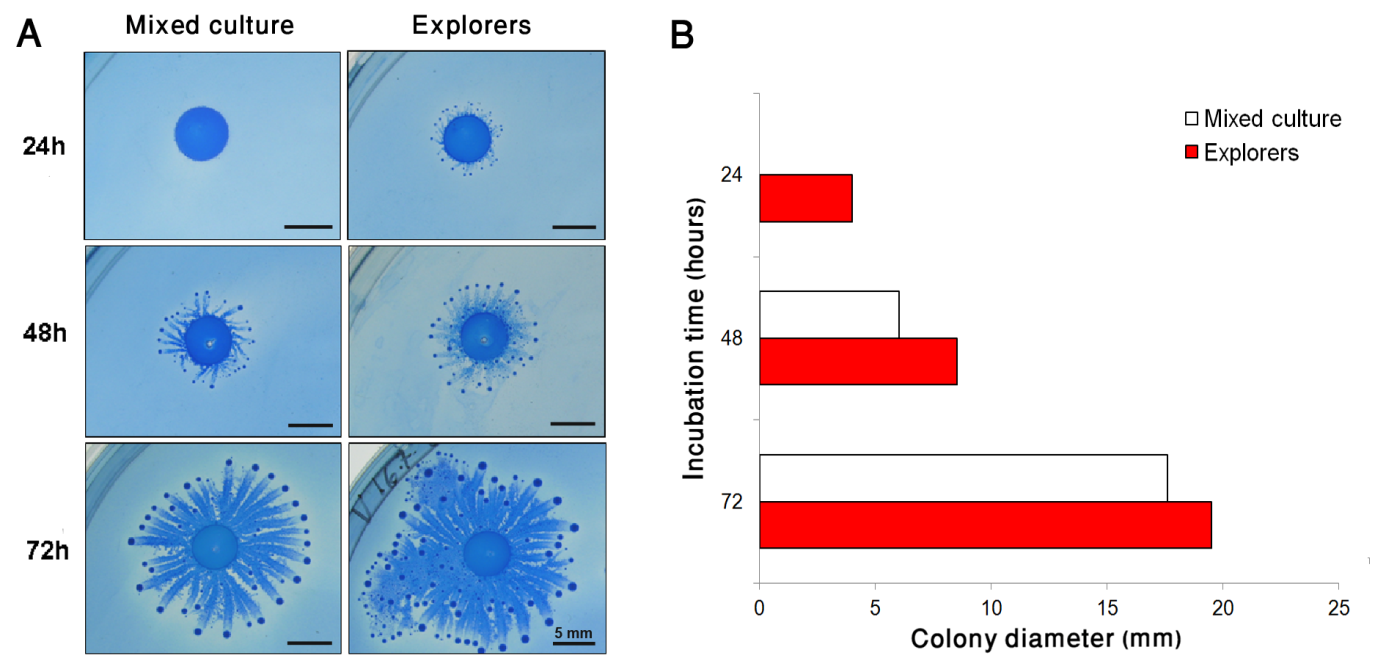


**Fig. S1.** Kinetics of colony development on peptone agar**.** *P. vortex* was grown for 18 h in liquid LB with and without 10 µg/ml kanamycin. Cells were then inoculated on peptone plates, (20 g/l, 2.25% w/v agar) and grown in 30°C for 3 days (A). Pictures were taken after 24, 48 and 72 h. Scale bar = 5 mm. Colony diameter (branches width, mm), was measured after each time point (B).

**S2. Susceptibility of *builders* to kanamycin**

**
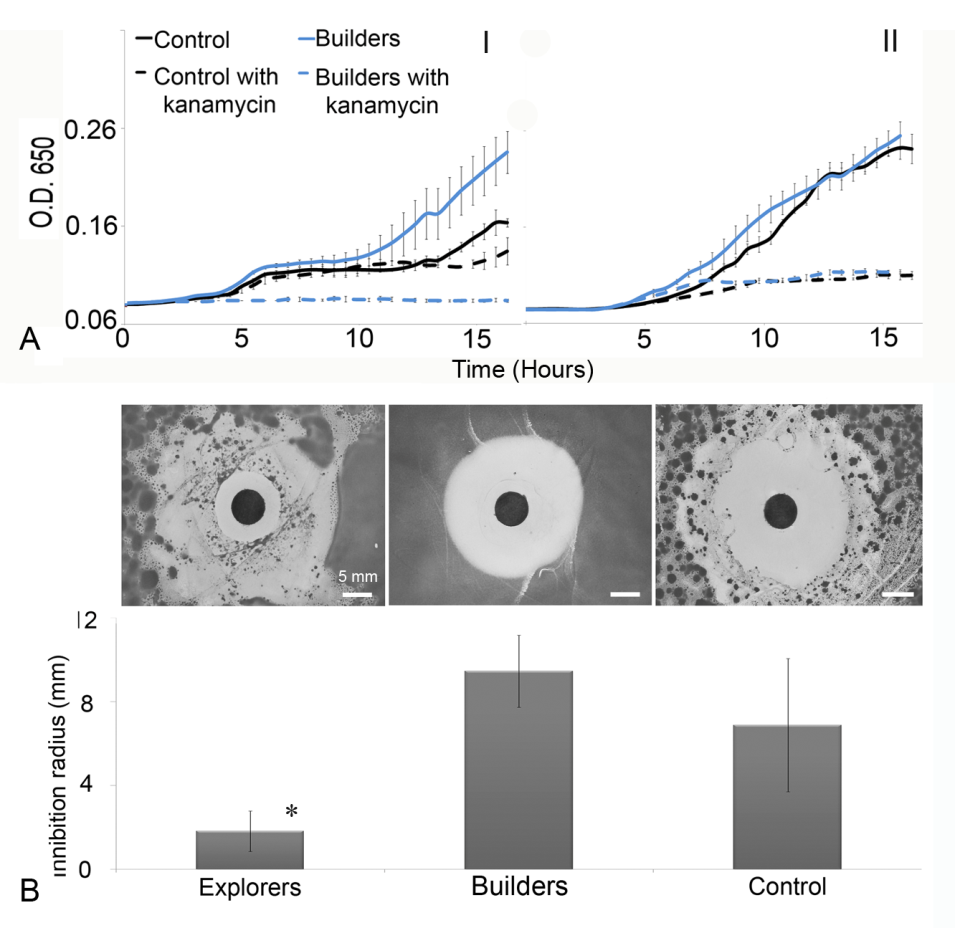
**

**Fig. S2**. *Builders* susceptibility to kanamycin. *Builders’* enriched culture, *explorers* and mixed culture were examined for resistance to kanamycin using disk diffusion assay. The bars represent the averaged values of inhibition radius. A significant difference is found between *builders’* and *explorers’* inhibition radius with *P* <8.6x10^-07^. Pictures on top of each bar represent the inhibition zone of each morphotype respectively. Scale bar – 5mm.

**S3. Identification and quantification of *explorers* in the culture**

**
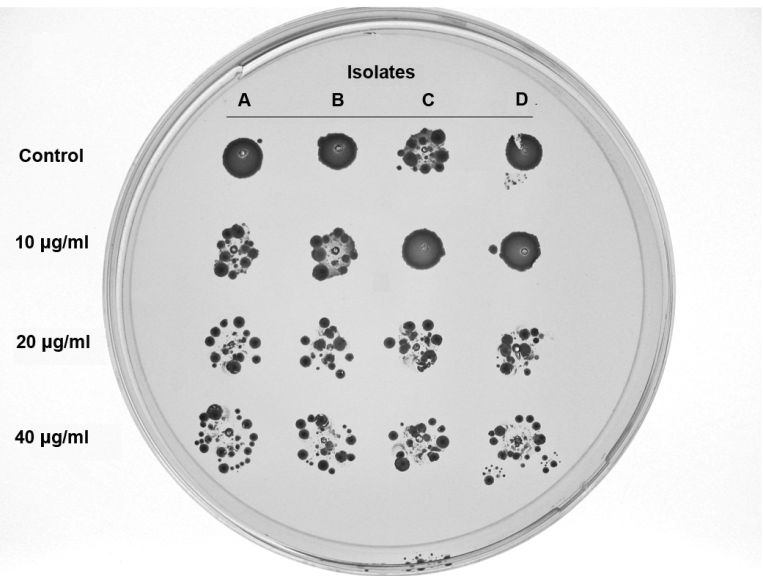
**

**Fig. S3. Identification of *explorers*. Cells isolated from 10, 20, 40 µg/ml kanamycin were spread over LB agar. Microcolonies were picked, re-inoculated on LB agar, and the expansion diameter was examined. An example of swarming assay of 4 isolated samples after 24 h incubation on LB plates. The expansion diameter (mm) presented in table S1A.**

| A |  |  |  |  |  |  |
| --- | --- | --- | --- | --- | --- | --- |
|  | **A** | **B** | **C** | **D** | **Average** | **SD** |
| **0** | 6.00 | 6.00 | 7.50 | 6.00 | **6.38** | 0.75 |
| **10** | 7.50 | 7.50 | 6.00 | 6.00 | 6.75 | 0.87 |
| **20** | 9.38 | 9.38 | 8.63 | 7.50 | 8.72 | 0.89 |
| **40** | 10.88 | 9.38 | 9.38 | 12.75 | 10.59 | 1.60 |
| B |  |  |  |  |  |  |
|  | **A** | **B** | **C** | **D** | **Average** | **SD** |
| **0** | 0.28 | 0.25 | 0.17 | 0.23 | **0.23** | 0.04 |
| **10** | 0.17 | 0.18 | 0.28 | 0.24 | 0.22 | 0.05 |
| **20** | 0.17 | 0.18 | 0.16 | 0.19 | 0.18 | 0.01 |
| **40** | 0.18 | 0.18 | 0.18 | 0.18 | 0.18 | 0.00 |

**Table S1.** Detection of *explorers* in the culture (A) Colony expansion diameter (mm) of isolated *explorers* (Fig. S3). (B) The absorbance at O.D._650_ of the same isolates, after 20 h of growth. Isolates that have shown 10% increase in colony diameter (A) and over 10% decrease in the absorbance after 20 h (B) were defined as *explorer.* The values of each isolate were compared to the average value of the control (bold). Isolates with a colony diameter value > 7.01 and O.D < 0.207 were identified as *explorers* (marked in gray). Evaluation of *explorers’* percentage was determined by division of the number of samples identified as *explorers* by the total number of samples.

**S4. Kanamycin challenge of *Builders***

**
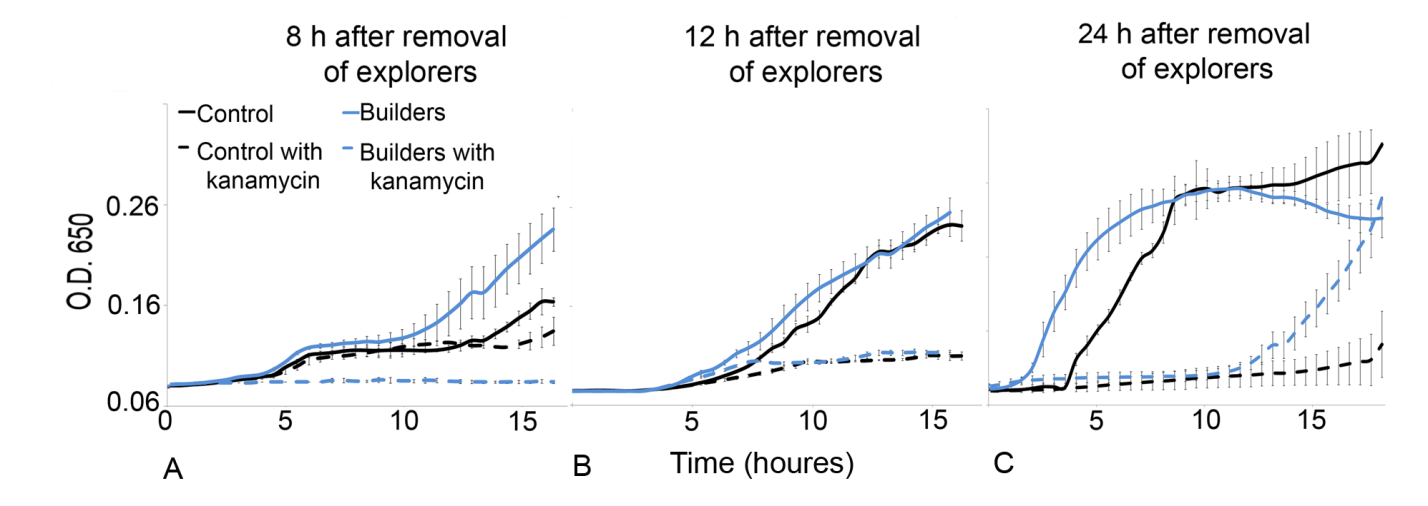
**

**Fig. S4.** Growth of *Builders’* enriched culture in the presence of kanamycin. *Builders*’ enriched culture (blue) obtained after X 4 transfers at the early log phase, were grown in liquid LB for 8 h (A), 12 h (B) and 24 h (C). After each time point samples were spread over LB agar then microcolonies were immediately picked and added into liquid LB with (dashed line) and without (smooth line) 20 µg/ml kanamycin and growth curves were examined. Mixed culture grown to the same time points were taken as a control (black). (A) After 8 h, the *builders* did not grow in the presence of kanamycin compared to mixed culture. (B,C), After 12 and 24 h, the culture had the same growth kinetics as the mixed culture. Suggesting *builders* are stable up to 8 h but revert to the mixed culture composition by 12 h.

**S5. Gene expression of *P. vortex* under exposure to kanamycin**

**Microarray Experimental Procedures**

**RNA Extraction RNA was preserved in 0.5 ml of RNAlater (Ambion,** Carlsbad, CA, USA**) and** extracted using Qiagen’s RNeasy minikit (Qiagen, Valencia, CA, USA). The RNA samples were examined using Bioanalyzer and found to be suitable for microarray analysis, by criteria of OD260/280 >1.8 and <2.2; OD260/230>1.0 and <2.4.

**Microarray hybridization** was carried out at Genotypic Inc. (Bangalore, India), samples were labeled using the Agilent’s Quick-Amp labeling Kit (Agilent Technologies Inc., Santa Clara, CA, USA) and QC was performed using Nanodrop (Thermo Fisher Scientific, Wilmington, DE 19810 USA). The cDNA samples were hybridized to Agilent *P. vortex* 8x15k array using the Agilent *in situ* Hybridiztion kit 5188-5244.

**Microarray analyses** The normalization was performed as follows. First, Cy3 and Cy5 signals were normalized on each chip separately providing the same distributions of the array's Cy5 and Cy3 log-signals. It was done for accurate estimation of the per array treatment-control differentiations. On the next stage, all signals across all arrays were normalized. Normalized log signals (Natural log, based on “e”) of “control” and “kanamycin” duplicates (CAT1, CBT1 KAT1, KCT1) were averaged and the difference between the averages was calculated. Up-regulated genes considered ones whose expression ratio was above 1.5 (log C>0.4). The genes were filtered based on presence of the kanamycin induced up-regulation and divided according to functional categories annotated by TIGR. The data is published in NCBI's Gene Expression Omnibus [[GSE35271](http://www.ncbi.nlm.nih.gov/geo/query/acc.cgi?acc=GSE35271)]

To understand the mechanisms responsible for the increased swarming capabilities of *explorers* enriched under growth with kanamycin, gene expression for both populations was further tested by microarray chip experiment. Microbes were harvested after 18 h incubation with 10 μg/ml kanamycin (this concentration doesn't significantly effects growth rate, though it is high enough to impact pattern formation). The normalized signals were analyzed and divided into functional categories (Fig. S5). Increased transcription levels were detected in gene expression profile of protein synthesis, purines and pyrimidines synthesis, protein fate and detoxification genes (i.e. functional groups as described by TIGR). Notably, about 60% of chemotaxis and motility functional group were over expressed by more than x 1.5 fold (Table S2. presents the lists the genes in this group that were upregulated). Most of these genes belong to class II flagella genes (e.g. *fliM, fliQ, flgD*). Others belong to the chemotaxis group and include the sensory transduction and components of two component phosphorylation systems *cheA*, *cheW*, *cheY,* as well as several methyl-accepting chemotaxis receptor genes. It is important to mention that genes from class III such as *flagellin*, *flgK*, *motA* and *motB* were induced but their averaged expression ratio was below the threshold so they were not mentioned at this list. Over-expression of flagella proteins is likely to explain the increased colony spreading and swarming motility of *explorers*.


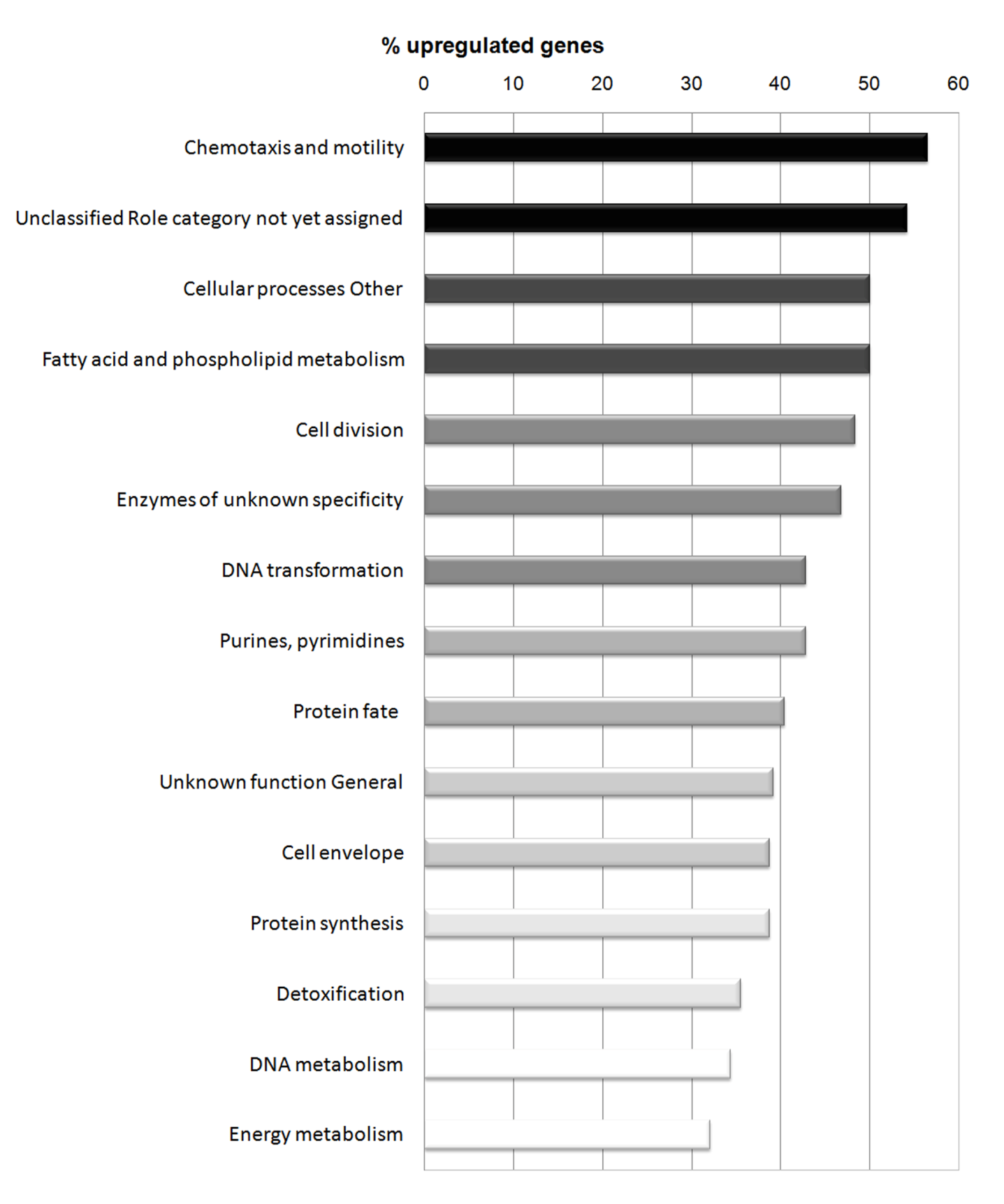


**Fig. S5.** Gene expression of *P. vortex* under kanamycin exposure. Gene expression profile of *P. vortex* culture after exposure to kanamycin. Normalized log signals (Natural log, based on “e”) of “control” and “kanamycin” duplicates were averaged and the difference between the averages was calculated. Up-regulated genes are all the genes whose expression ratio is above 1.5 (log C>0.4). The genes were filtered based on presence of the kanamycin induced upregulation and divided according to functional categories annotated by TIGR. The bars represent the percentage of upregulated genes from total number of genes at each category.

**Table S2.** Expression of flagella and chemotaxis genes under exposure to kanamycin. All genes represented in the table have expression ratio above 1.5 (log C>0.4). Genes’ IDs were represented by the Query Locus Tag, published in NCBI’s GEO [GSE35271].

**S6. Analysis of flagella genes expression using Genome Holography method**

In order to chose a representative flagella genes for examination with real-time PCR we decided to analyse the microarray results using Genome Holography (GH) method. The GH method was applied for analysis of gene networks (Madi *et al.,* 2008). The GH method includes collective normalization of the correlations, dimension reduction algorithms (PCA) of the matrices of normalized correlations, and location in a reduced 3-dimentinal space whose axes are the three leading principal vectors of the PCA ([Roth *et al.,* 2011](#_ENREF_1)). For GH analysis we used the normalized log-signal of 8 samples after 18 h growth: 3 control repetitions; 3 kanamycin repetitions; and 2 mitomycin C repetitions. The GH Analysis showed that after 18 h under antibiotics the genes assembled mainly into two clusters (Fig. S6). In the first cluster there are class II flagella genes. Cluster 2 contains the class III flagella genes, *motA, flgK* and *flagellin.*

The GH results indicate that under antibiotic treatment, the transcription of the flagella genes is separated into 2 functional groups, thus a selection of several genes from each group for gene expression examination would represent the transcription of the whole flagella complex.


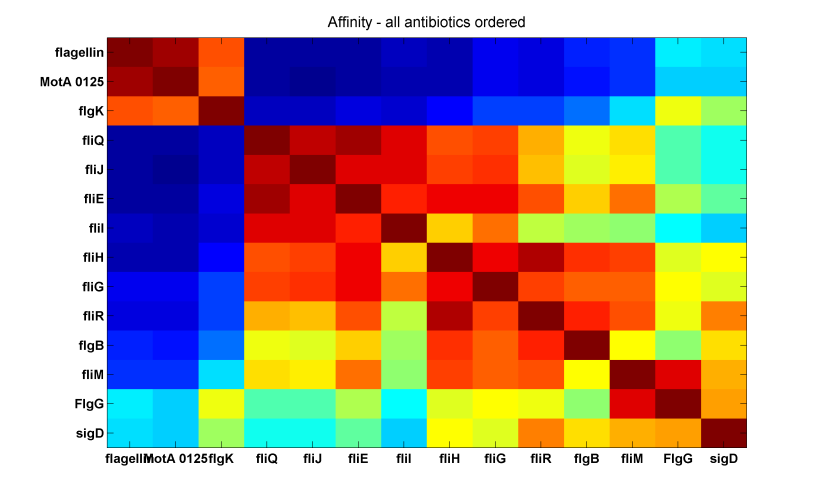

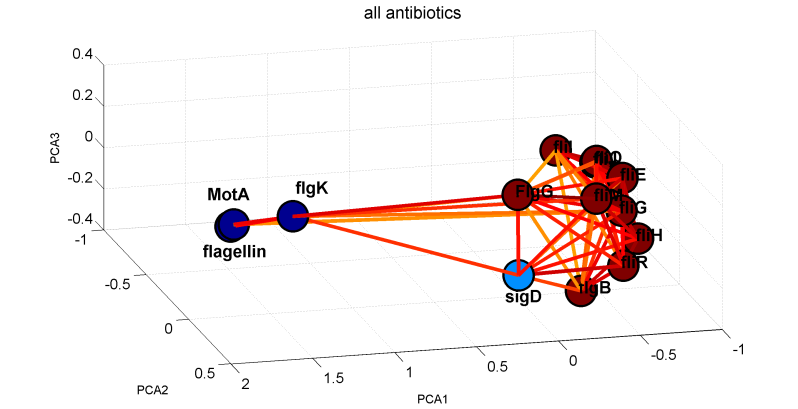


**Fig. S6.** Holographic network of *P. vortex* flagella genes. Holographic network of flagella genes after 18 h of exposure to antibiotics. The correlation lines connecting the genes were set by correlation threshold greater than 0.7 and smaller than -0.4. The matrices sorted by dendrogram algorithm.

**S7. Flagella genes, used for RT-PCR**

| Number | * Name | Primer sequence (5'-3') |
| --- | --- | --- |
| 1 | NT01MV5021_F | GACCCTGACTACTTCTGATGCAGAT |
| 2 | NT01MV5021_R | GCTGTCGATTTGAGCAGACAAT |
| 3 | NT01MV2405_F | ACTGATGGCGGTGTACAAGGA |
| 4 | NT01MV2405_R | GGCATTCGAGCCTTCTTGATA |
| 5 | NT01MV5015_F | CAGACCACGCGCTTGGTT |
| 6 | NT01MV5015_R | CGCTGCATTCCTGGAAAAG |
| 7 | NT01MV5063_F | TTCTCCCAGGCGGGTATG |
| 8 | NT01MV5063_R | GCCGATAACGGCTCCAAGTA |
| 9 | NT01MV5013_F | GCGGAGAAAATTCAGCGTTT |
| 10 | NT01MV5013_R | CGACGGGCACCTCATAAGTAC |
| 11 | NT01MV2417_F | CGGAGATGTCATTTCATTGAACA |
| 12 | NT01MV2417_R | CGCTGCATTCCTGGAAAAG |
| 13 | NT01MV2435_F | TGACGTGACCGAGAAGGAGTT |
| 14 | NT01MV2435_R | CAGTGTCATGACCGCCACTT |
| 15 | 16S_F | CAACGCGAAGAACCTTACCAA |
| 16 | 16S_R | GTCCCGAAGGCCGCTACTAT |

**Table S3**. Primers for flagella genes, used for RT-PCR. * Genes’ IDs were represented by the Query Locus Tag, published in NCBI’s GEO [GSE35271].

| Number | * Name | Common name | Gene symbol |
| --- | --- | --- | --- |
| 1 | NT01MV5021 | flagellin | hag |
| 2 | NT01MV2405 | flagellar protein export ATPase FliI | fliI |
| 3 | NT01MV5015 | flagellar hook-associated protein FlgK | flgK |
| 4 | NT01MV5063 | chemotaxis protein MotA (Motility protein A) | motA |
| 5 | NT01MV2435 | RNA polymerase sigma-D factor (Sigma-28) | sigD |

**Table S4.** Annotation of hypothetical genes.

**S8. *Explorers’* hyperflagellation**


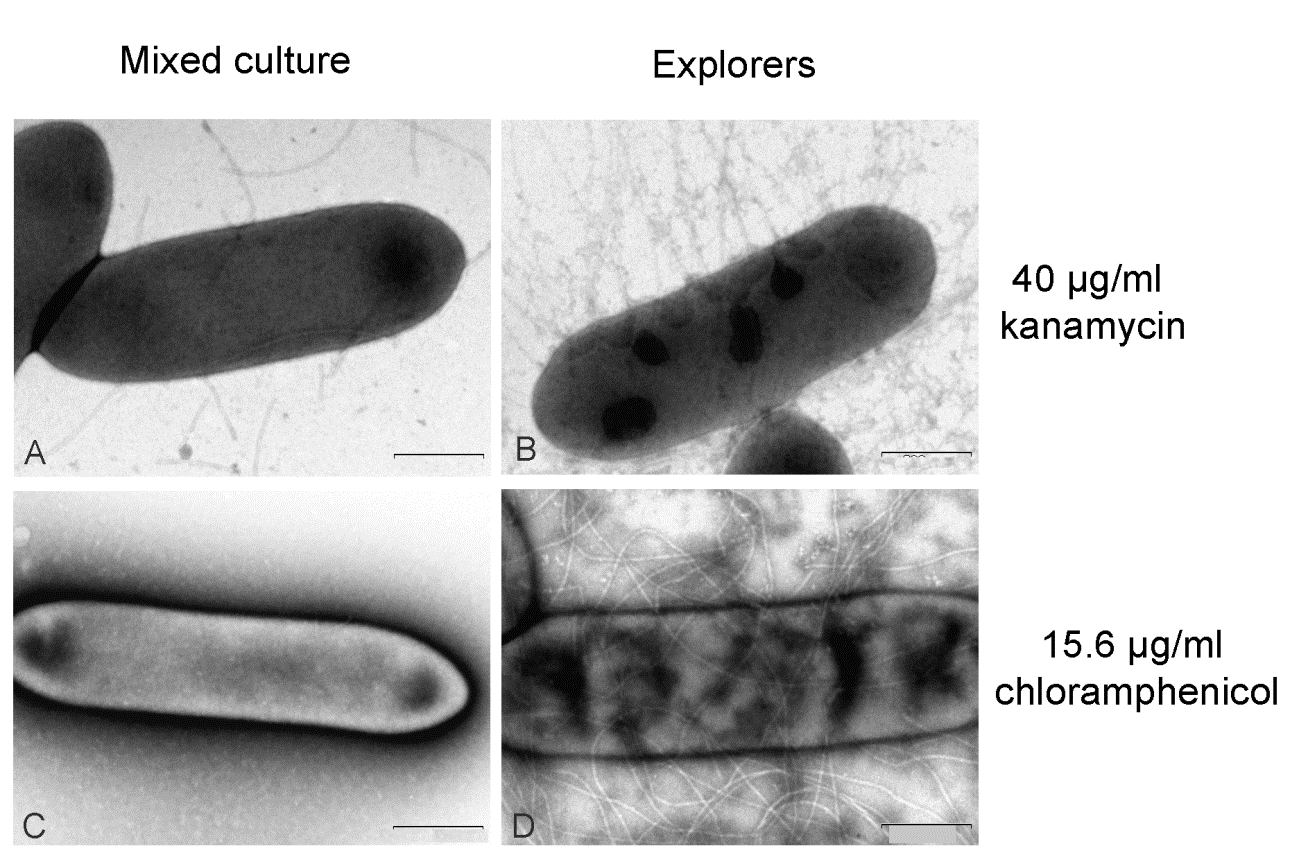


**Fig. S7.** Increase of flagella filaments number in *explorers* isolated from 40 µg/ml kanamycin (A,B) or 15.6 µg/ml chloramphenicol (C,D). A,C Mixed culture. B,D *explorers*. Magnification of X 50K. Scale bar = 500 nm.

**S9. Pre-exposure to various antibiotics**

To examine if the tolerance of *explorers* is restricted to aminoglycosides (kanamycin) or is a general phenomena, the culture was pre-exposed to various antibiotics. Table S5 represents the list of tested antibiotics. The translation inhibitors are represented by kanamycin, spectinomycin (aminoglycosides family), and chloramphenicol (chloramphenicolum group). DNA gyrase inhibitor is represented by novobiocin ([aminocoumarin](http://en.wikipedia.org/wiki/Aminocoumarin)s family), the transcription inhibitor is represented by rifampicin (rifamycin group) and the inhibition of cell wall synthesis is represented by ampicillin from penicillin group of beta-lactam.

| **Antibiotic** | **Kanamycin** | **Spectinomycin** | **Chloramphenicol** | **Novobiocin** | **Rifampicin** | **Ampicillin** |
| --- | --- | --- | --- | --- | --- | --- |
| **Mode of action** | Affecting 30S ribosomal subunit and causing a frame-shit | Binds to the 30S ribosomal subunit | Inhibiting the enzyme peptidyl transferase, which inhibits ribosomal activity and protein synthesis | Inhibiting DNA gyrase | Inhibiting RNA polymerase preventing transcription of messenger RNA | Competitive inhibitor of the enzyme transpeptidase |
| **Type** | aminoglycosides | aminocyclitol antibiotic, closely related to the aminoglycosides | bacteriostatic antimicrobial | [aminocoumarin antibiotic](http://en.wikipedia.org/wiki/Aminocoumarin) | [rifamycin group](http://en.wikipedia.org/wiki/Rifamycin) | beta-lactam antibiotic |
| **MIC µg/ml** | 62.5 | 3.15 | 7.8 | 0.009 | 0.031 | 3.15 |
| ***Explorers* emergence** | + | + | + | + | + | + |
| **Concentration**  **of *explorers* isolation µg/ml** | 31.25 | 3.15 | 7.8 | 0.009 | 0.031 | 1.5 |
| **Time until appearance on plate (hours)** | 8 | 8 | 24 | 18 | 24 | 8 |
| **Hyper motility** | + | + | + | + | + | + |
| **Phenotype stability*** | + | + | + | + | - | + |

**Table S5.** Emergence of *explorers* under different antibiotics.

*Stability of the phenotype was examined 24 h after isolation on LB plate.

**S10. Assays for specie identification**

To insure that the stable morphotype *explorers,* is not co-existing specie, two genomic identification assays have been made: 16SrRNA sequencing and RAPD-PCR (Welsh and McClelland, 1990; Williams *et al*., 1990).

**16SrRNA:** *Explorers’* microcolonies were picked, grown in liquid LB medium for 18 h. Genomic DNA was extracted with "DNA purification kit" (Promega, Madison, WI, USA). DNA concentrations were quantified using a NanoDrop (Thermo Fisher Scientific), and normalized to 10 ng/µl in 1× TE for direct use in PCR. Universal bacterial primers, 5′-AGAGTTTGATCCTGGCTCAG-3′, 5′-TACGGCTACCTTGTTACGACTT-3′ were used in PCR. Thermocycling parameters were: 95°C 1.5 min, (95°C 45 sec, 52°C 45 sec, 72°C 90 sec) x 34 cycles. 16SrRNA sequences were then identified using BLAST against the complete GenBank nucleotide database (http://www.ncbi.nlm.hih.gov) and JGI against the bacterial nucleotide database (<https://img.jgi.doe.gov/cgi-bin/er/main.cgi>). The 16sRNA sequencing has shown 97% identity to *P. vortex* genome.

649989979.NZ_ADHJ01000002 Paenibacillus vortex V453 CNT_PVOR1000002, whole genome shotgun sequence.

Length = 1545

Score = 1568 bits (791), Expect = 0.0

Identities = 850/868 (97%), Gaps = 4/868 (0%)

Strand = Plus / Plus

**RAPD-PCR:** The assay utilizes a single arbitrary primer (16srRNA_F 5′-AGAGTTTGATCCTGGCTCAG-3′, SPOVFA_F 5'-GCACCGGGATGACGATTGCCCGTA-3’) under permissive PCR conditions. 10 ng DNA, extracted from *explorers’* and mix cultures were used as template. The PCR amplification protocol was as follows: (94°C 5 min, 40°C 5 min and 70°C 5 min) x 4 cycles followed by (94°C 1min ,55°C 1 min, 70°C 2 min) x 30 cycles with a final primer extension at 70°C for 5 min. RAPD-PCR products (fingerprinting) have shown that *explorers’* morphotype is the same species as *P. vortex* (mixed culture) (Fig. S8).


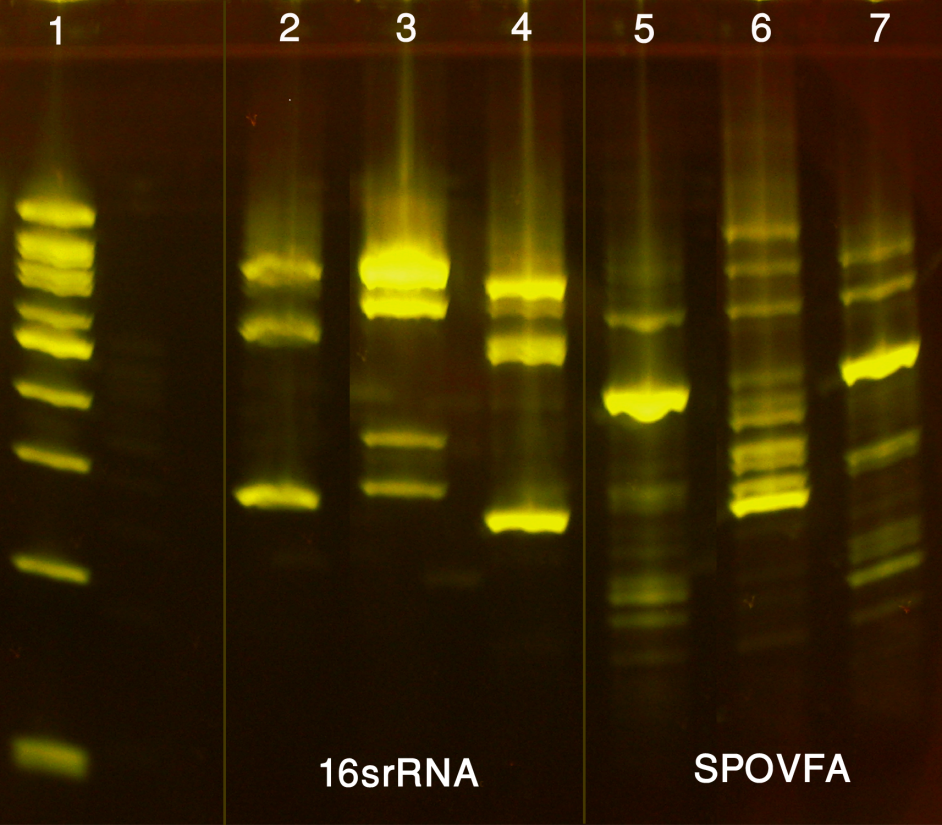


##### Fig. S8. RAPD-PCR of *explorers*. Columns 1 – 100bp Marker (Talron Biotech LTD, Rehovot. Israel). Columns 2,5 – *Explorers.* Columns 3,6 – *B. subtilis* 168 (negative control). Columns 4,7 – *P. vortex* (mixed culture). Primers for amplification - 16Sr*RNA* (2-4), SPOVFA (5-7). Amplification profiles of *explorers* and *P. vortex* were similar under both primers in contrast to *B. subtilis* 168, indicating that *explorers* and *P. vortex* are the same species.

**References**

Madi, A., Friedman, Y., Roth, D., Regev, T., Bransburg-Zabary, S., Ben-Jacob, E. (2008) Genome Holography: Deciphering Function-Form Motifs from Gene Expression Data. *PLoS ONE* **3**: e2708.

Roth, D., Madi, A., Kenett, D. Y., Ben-Jacob, E. (2011) Gene Network Holography of the Soil Bacterium Bacillus subtilis. In *Biocommunication in Soil Microorganisms*. Witzany, G. (ed). Berlin Heidelberg: Springer pp. 255-281.

Welsh, J., and McClelland, M. (1990) Fingerprinting genomes using PCR with arbitrary primers. *Nucleic Acids Res* **18**: 7213-7218.

Williams, J.G., Kubelik, A.R., Livak, K.J., Rafalski, J.A., Tingey, S.V. (1990) DNA polymorphisms amplified by arbitrary primers are useful as genetic markers. *Nucleic Acids Res* **18**: 6531-6535.
